# Building Artificial Neural Networks for the Optimization of Sustained-Release Kinetics of Metronidazole from Colonic Hydrophilic Matrices

**DOI:** 10.3390/pharmaceutics17111451

**Published:** 2025-11-10

**Authors:** Cristina Maderuelo, Roberto Arévalo-Pérez, José M. Lanao

**Affiliations:** 1 Area of Pharmacy and Pharmaceutical Technology, Department of Pharmaceutical Sciences, Faculty of Pharmacy, University of Salamanca, 37007 Salamanca, Spain; cmaderuelo@usal.es (C.M.);; 2 Institute of Biomedical Research of Salamanca (IBSAL), 37007 Salamanca, Spain

**Keywords:** artificial neural network, computational prediction, in vitro modelling, colonic drug delivery, pharmaceutical formulation, controlled release systems, artificial intelligence

## Abstract

**Introduction**: Drug development has traditionally used mathematical models to predict formulation behavior. **Objective**: Building artificial neural networks for the drug release evaluation of drug delivery systems using sustained-release metronidazole-coated colonic hydrophilic matrices as a model. **Methods**: The technological factors associated with the biopharmaceutical performance of hydrophilic metronidazole matrices were evaluated using a quality by design approach (QbD). The developed neural network includes variables related to the technological process for producing the matrices. These are related to the materials used, such as the type and viscosity of core polymers, the type of coating agent, or the matrix production process, such as the mixing time of core materials or the percentage of the coating agent. The output variables of the neural network were the percentages of drug released in vitro at 1, 6, 12, and 24 h and the mean dissolution time of the matrix. An iterative quasi-Newton method was used to train the artificial neural network. **Results**: A neural network with excellent prediction capacity allows selecting the technological variables with the greatest influence on the % of drug dissolved: the type of coating agent used and the percentage of the total weight increase after coating for 1 h and 6 h of drug release and also the viscosity of the HPMC for 12 and 24 h. **Conclusions**: The optimized neural network demonstrated an excellent predictive capacity for in vitro drug dissolution profiles, allowing the use of this type of methodology based on artificial intelligence methods in the optimization of drug delivery systems.

## 1. Introduction

The artificial neuron is a mathematical model inspired by the biological neuron in order to simulate complex systems [1,2]. A neural network consists of interconnected neurons organized into layers [3,4]. The learning process of the network, commonly referred to as deep learning, is achieved either by adapting the network architecture or by optimizing the synaptic weights through iterative algorithms such as Newton’s method and related approaches [5,6,7,8]. Currently, there are various applications of neural networks in pharmaceutical technology and, more specifically, in the optimization of controlled-release systems [4,5,6,7,8,9]. Artificial neural networks (ANNs) have proven to be powerful tools in the design of dosage forms [10]. ANNs have been used to optimize transdermal formulations and also to predict the skin permeation of diclofenac from topical patches [11,12]. Both studies demonstrate how ANNs can reduce the number of experimental trials required and improve efficiency in dosage form development.

The effective treatment of local colonic diseases, such as diverticulitis, requires targeted drug delivery to the infection to reduce systemic side effects. Metronidazole is absorbed in the upper gastrointestinal tract. Matrix tablets with hydrophilic polymers combined with a functional coating layer can be used to overcome these limitations [13]. Given the complexity in optimizing the drug release behavior of multi-component pharmaceutical systems, the use of mathematical modeling tools, such as artificial neural networks, becomes highly valuable.

For controlled-release systems, decision trees and Elman dynamic neural networks were used to design matrix tablets, achieving accurate predictions of release profiles [14]. Complementarily, combined experimental design and ANNs have been used to study implantable matrices, allowing not only the prediction of release rates but also a deeper understanding of release mechanisms [15]. Moreover, mathematical models for hydrogels highlight that their integration with tools such as ANNs can improve the design of advanced delivery systems [16]. These experiments contribute to highlighting the interest of ANNs in drug discovery and emphasize their role in drug delivery systems [17,18].

The aim of this research is to build an artificial neural network for the optimization of the drug release of drug delivery systems using model hydrophilic matrices coated for the colonic release of metronidazole developed under a quality by design (QbD) approach. This study integrates experimental formulation data generated under a quality by design (QbD) framework with advanced computational modeling. This approach introduces a novel predictive tool for guiding formulation optimization and enhancing control over colonic drug delivery kinetics.

## 2. Materials and Methods

### 2.1. Hydrophilic Matrices for the Colonic Release of Metronidazole

To evaluate the different factors involved in the biopharmaceutical behavior of hydrophilic matrices for the colonic delivery of metronidazole, a quality by design (QbD) approach, based on Failure Mode and Effects Analysis (FMEA), was employed to develop a 2^5^ fractional factorial design of experiments (DoE), incorporating a central point and replicates, leading to the manufacture of 28 batches of coated matrix tablets [13]. The five factors identified through FMEA were tested at two levels each: HPMC grade (K15, K35), HPMC/Chitosan ratio (1:3, 3:1), mixing time (10, 20 min), coating agent (Eudragit^®^ RL 30D, Eudragit^®^ FS 30D), and percentage increase in total weight after coating (%ΔW; 10%, 20%). All the matrix tablets contained 66.69% metronidazole, 0.50% Aerosil200 VV Pharma, and 1.00% glyceryl behenate. The remaining 31.81% was distributed between HPMC and CH in ratios of 3:1, 1:3, or 1:1 according to the DoE. All components were sieved through a 0.315 mm mesh and blended at 35 rpm in an Erweka biconic rotating drum (Erweka^®^, Langen, Germany). The lubricant was subsequently added and mixed for 5 min. The final blend was then directly compressed into matrix tablets using an 11 mm round punch on a Bonals^®^ BMT press (Barcelona, Spain), with a target weight of 750 mg and a hardness range of 90–140 N. The coating process utilized two functional polymers (Eudragit RL30D and FS30D) and was carried out in a rotating pan at 35 rpm, maintaining the bed air temperature at 40 °C during application and followed by a 2 h curing step at 40 °C.

All manufactured metronidazole-coated matrix tablets were subject to physicochemical control (e.g., weight variation, hardness, and friability) to confirm compliance with the specifications previously established in a QTPP defined by the QbD methodology. These processes are fully documented in a previous publication [13]. These 28 batches constitute the basis of the dataset for the development, training, and validation of the current neural network modeling.

### 2.2. Building the ANN for Metronidazole Colonic Release Matrices

The building of the neural network for a drug delivery system (DDS), such as metronidazole colonic release matrices, involves the following steps.

1. Selection of the input variables. In the case of DDSs, the input variables correspond to critical material attributes (CMAs) or critical process parameters (CPPs) involved in the technological development of the DDS, previously selected through a risk assessment process.
2. Definition of the output variables. In DDSs, the output variables correspond to the response of the system and are usually related to their biopharmaceutical and pharmacokinetic behavior, usually the percentage of drug dissolved using in vitro dissolution tests or the degree of absorption of the drug evaluated by in vivo studies.
3. Definition of the number of layers in the neural network, the number of neurons in each layer, and the activation function that will be used in each of the layers in the neural network.
4. Neural network training. The training or learning of the artificial neural network can be carried out in two ways. A. Through the optimization of synaptic weights by means of an iterative algorithm, usually based on Newton’s method, which minimizes the error function. B. Modifying the architecture of the neural network.
5. Validation of the neural network. Evaluation of the predictive performance.

**Figure 1:**
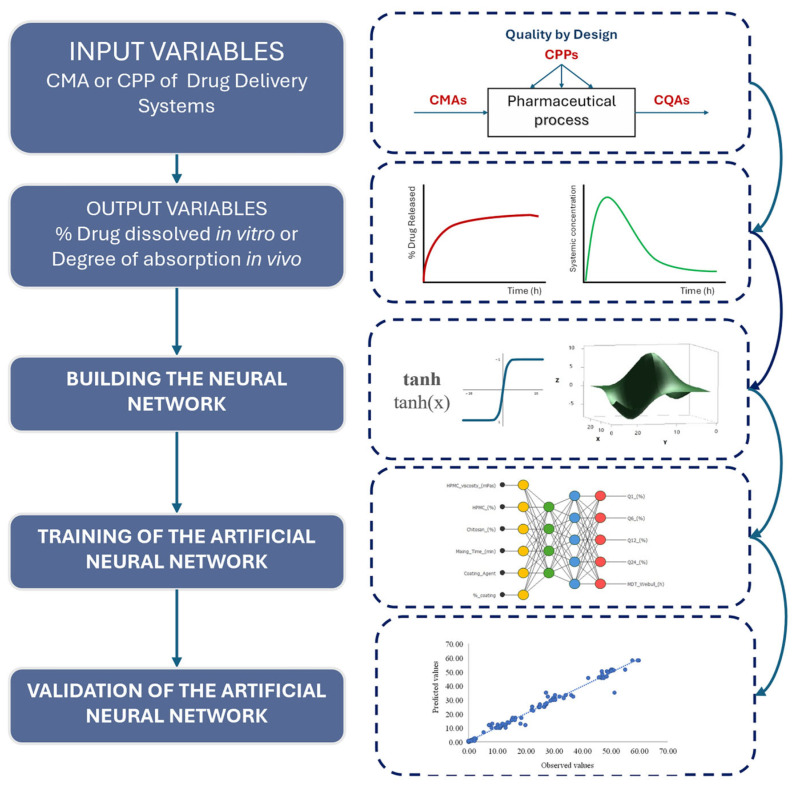
Flowchart for building and implementing a neural network for drug delivery systems.

Figure 1 presents the flowchart illustrating the sequential stages involved in building an artificial neural network for drug delivery systems. This flowchart was applied for the drug release evaluation of colonic metronidazole delivery matrices.

### 2.3. Input and Output Variables

The selection of input variables for the network was guided by a preceding Risk Analysis and Design of Experiments (DoE), which identified formulation and process factors with a potential impact on the Critical Quality Attribute (CQA) dissolution release of the metronidazole colonic tablets. This methodology section defines the modeling variables; however, the original experimental data (input/output values) and their full characterization are referenced from a prior published work [13]. The input variables tested in the neural network were HPMC viscosity grade (mPa.s), HPMC (%), Chitosan (%), mixing time (min), coating agent, and % total weight increase after coating.

The output variables tested in the neural network were the amounts of metronidazole released from the matrices at different times, Q1 (%), Q6 (%), Q12 (%), and Q24 (%), and the mean dissolution time MDT (h) optimized using the Weibull equation. The dissolution methodology used buffering 7.2 pH media, since the acid resistance of the enteric coating had already been validated by a disintegration test in 0.1 M HCl on the worst-case batches. Moreover, the detailed kinetic analysis of the metronidazole release profiles, which is part of the modeling approach, was previously reported. This analysis, primarily conducted using the Weibull model, confirmed a sustained-release pattern, with MDT in the majority of cases exceeding 24 h. Both the MDT and the lag time are influenced by the formulation variables (specifically polymer concentration and coating type) [13].

The resulting experimental batches (F1–F28) from the design of experiments thus represented combinations of excipient grade, proportion, processing time, and coating conditions. These were used to provide input variables for the neural network optimization aimed at the formulation performance.

**Figure 2:**
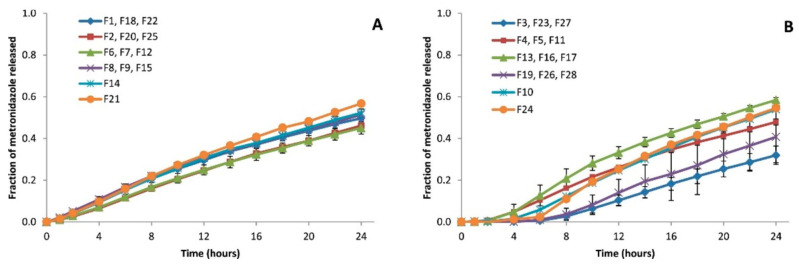
Dissolution profiles of metronidazole from sustained-release hydrophilic matrices at 7.2 buffering media. (**A**) Batches coated with Eudragit^®^ RL 30D (time-dependent coating); (**B**) batches coated with Eudragit^®^ FS 30D (pH-dependent coating) [13]. Reproduced with permission.

Figure 2 shows the results of metronidazole dissolution profiles using the 28 batches of sustained-release matrices developed based on the previously described experimental design [13]. The plotted release profiles represent the mean ± SD of the corresponding replicate or central point batches. Tables S1-A and S1-B show the formulation differences between every replicate. Modifications in the dissolution profiles are observed depending on the type of coating. Comparing the dissolution profiles obtained with the time-dependent coatings (A) versus the pH-dependent coatings (B), the latter showed long latency times and greater inter-batch variability.

### 2.4. Computational Methodology for Building the ANN

An artificial neural network (ANN) based on an approximation model was developed to predict the amount of metronidazole released (Q(%)) from sustained-release coated tablets at different times and the mean dissolution time (MDT) derived from the Weibull model. The details for the computational methodology are the following:

#### 2.4.1. ANN Architecture

The architecture of the neural network consisted of three layers: a scaling layer, one hidden perceptron layer, and an unscaling layer.

#### 2.4.2. Data Partitioning

The dataset was automatically divided into three subsets to ensure generalization and avoid overfitting:

Training set: 60% of total data (364), used for weight adjustment during optimization. Selection (validation) set: 20% of data, used to monitor model generalization and determine early stopping. Testing set: 20% of data, used exclusively for final model evaluation. Data partitioning was randomized to maintain statistical representativeness across subsets.

#### 2.4.3. Data Preprocessing

Ensure numerical stability and improve convergence:

Input scaling method: Mean–standard deviation (standardization), where each input variable was transformed to have zero mean and unit variance. Output unscaling method: Minimum–maximum normalization, where the model predictions were transformed back to their original physical units for interpretability.

#### 2.4.4. Training Algorithm and Hyperparameters

The network weights were optimized using a quasi-Newton (BFGS) training algorithm, suitable for nonlinear regression problems.

Key hyperparameters included the following: Loss function: Mean squared error (MSE) between predicted and experimental outputs. Regularization method: L2 weight decay to prevent overfitting. Learning rate adaptation: Automatic adjustment by Neural Designer to balance convergence speed and stability. Initial weights: Randomized within a uniform distribution.

#### 2.4.5. Stopping Criteria

Training was automatically stopped according to Neural Designer’s built-in convergence conditions:

1. Minimum improvement in selection error: training stopped when validation error improvement was less than 10^−6^ over 100 consecutive epochs.
2. Maximum number of iterations: 10,000 epochs.
3. Gradient norm condition: optimization terminated if the gradient norm fell below 10^−9^.
4. Early stopping: triggered when selection error began to increase while training error continued to decrease, ensuring optimal generalization.

### 2.5. Model Selection and Training

For model selection, we analyzed the training error, which evaluates the neural network’s ability to fit the available data, and the selection error, which estimates its ability to generalize to new data. To achieve this, an incremental order strategy was applied, in which different network complexity configurations were evaluated, represented by the number of neurons in the hidden layer. The error function used was mean squared error (MSE). Based on the analysis of the errors associated with each configuration, it was determined that the optimal architecture corresponded to a network with four neurons in the hidden layer.

Once the architecture was defined, the artificial neural network was trained using a quasi-Newton iterative method, designed to minimize the overall error of the network. The training error was considered as the error of the AAN computed on the training dataset, and the selection error was considered as the error computed on a validation dataset. During training, both the training error and the selection error were continuously monitored in each iteration.

### 2.6. Neural Network Validation

An internal validation with the same data used to build the neural network was used. The predictive performance of the neural network was assessed using linear regression analysis and by overlaying the simulated amounts of metronidazole released with the observed data. The overall predictive performance of the artificial neural network was assessed in terms of bias and precision, using the ANN predictions as predicted values (PRED) and the experimental dissolution measurements as observed values (OBS) using the average-fold error (AFE) and absolute average-fold error (AAFE), respectively. Fold errors quantify the bias and overall predictive accuracy, respectively, in terms of fold deviation between predicted and observed values. An AFE value was regarded as acceptable when falling within a 2-fold error range (0.5–2-fold) [19]. Furthermore, these metrics were complemented with conventional absolute error measures, including the Root Mean Squared Error (RMSE) and Mean Absolute Error (MAE). Values for these were regarded as acceptable when falling below a predefined threshold of 10% of the mean observed value. Finally, the predictive capability of the ANN was benchmarked against the Weibull kinetic model and the Multiple Linear Regression model (MLR) previously performed [13]. The equations employed to calculate AFE, AAFE, MAE, and RMSE were as follows:

(1) $$\mathrm{AFE}=10^{\frac{1}{n}\sum \log(\frac{\mathrm{PRED}}{\mathrm{OBS}})}$$

(2) $$\mathrm{AAFE}=10^{\frac{1}{n}\sum |\log(\frac{\mathrm{PRED}}{\mathrm{OBS}})|}$$

(3) $$\mathrm{MAE}=\frac{1}{n}\overset{n}{\underset{i=1}{\sum }}\lceil \mathrm{OBS}-\mathrm{PRE}\rceil$$

(4) $$\mathrm{RMSE}=\sqrt{\frac{1}{n}\overset{n}{\underset{i=1}{\sum }}{\left(\mathrm{OBS}-\mathrm{PRE}\right)}^{2}}$$

where n represents the total number of experimental data points.

### 2.7. Neural Network Software

Neural Designer is a machine learning software tool specialized in the development, training, and deployment of artificial neural network models through high-performance numerical computing. It integrates advanced algorithms for supervised and unsupervised learning, including feedforward and deep neural networks. The software enables the construction of neural networks comprising multiple layers of nonlinear processing (Neural Designer, version 4) [20].

## 3. Results

### 3.1. Neural Network Modeling

#### Neural Network Architecture

**Figure 3:**
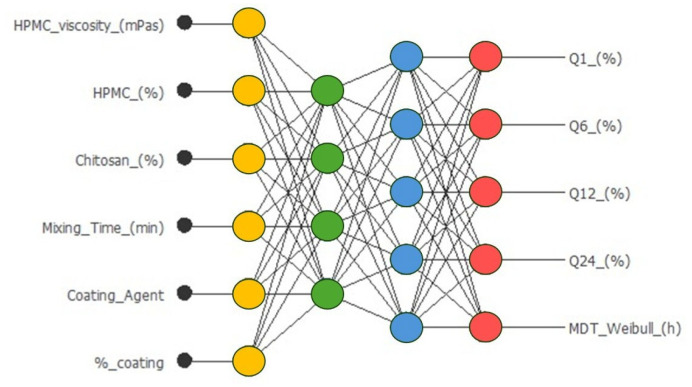
Neural network for the prediction of amounts of Q (%) of metronidazole released from sustained-release coated tablets at different times, and the MDT, mean dissolution time, from the Weibull. Equation: (Q(%)t = Q(%)max × (1 - exp(−(t−tlag) ^β/MDT)))). Input neurons (yellow), hidden neurons (green and blue, using a hyperbolic tangent function), and output neurons (red, using a linear function).

The architecture of the optimized neural network for metronidazole-coated matrices is shown in Figure 3.

Input layer: six neurons corresponding to formulation and process variables. Hidden layer: one layer with four neurons, employing the hyperbolic tangent activation function. Output layer: five neurons corresponding to the five response variables (Q (%) at different times and MDT), with a linear activation function. Total synaptic weights: 53. The complete architecture was therefore 6:4:5 (input/hidden/output).

### 3.2. Training Process

**Figure 4:**
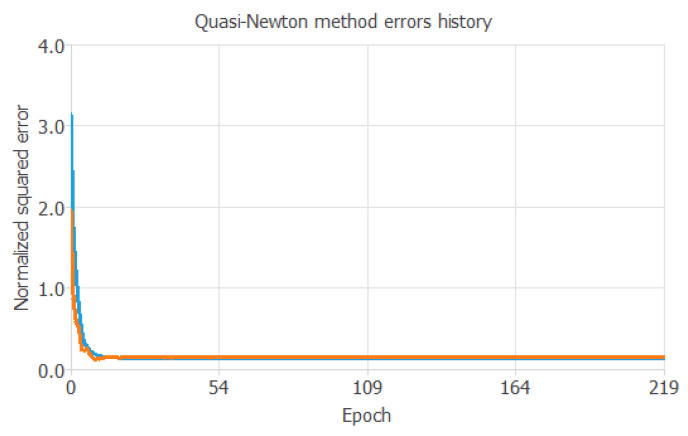
Evolution of the training (blue) and selection (orange) errors as a function of the iteration number.

Figure 4 shows the training and selection errors in each iteration. The blue line represents the training error, and the orange line represents the selection error.

### 3.3. Input and Response Variables

**Figure 5:**
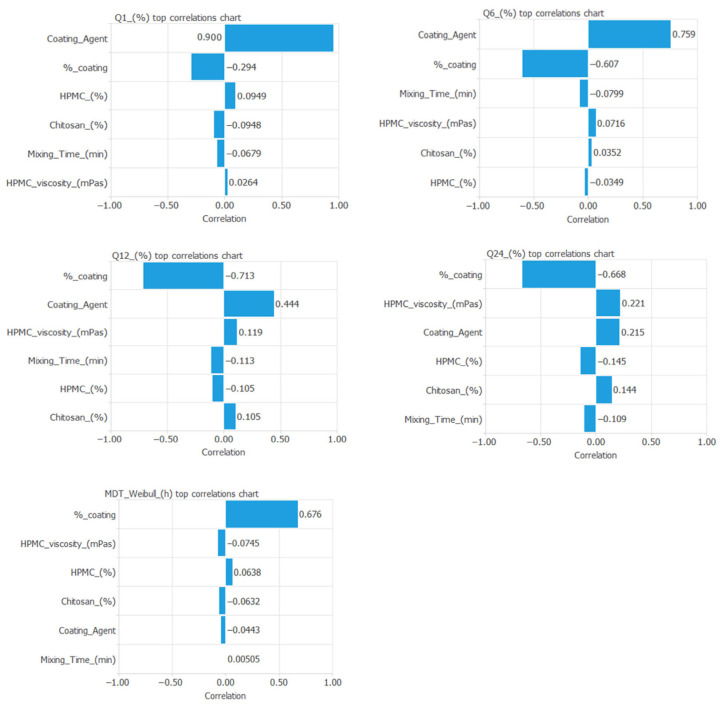
Correlation between the input and output variables included in the artificial neural network.

Figure 5 illustrates the correlation between the input and output variables incorporated into the neural network.

### 3.4. Error Statistics

The error statistics quantify the minimum, maximum, mean, and standard deviation of the discrepancies between the neural network-predicted outputs and the actual observed data. Table 1 presents the minimum, maximum, mean, and standard deviation of both the absolute and percentage errors of the neural network for the output variables.

**Table 1:** Minimum, maximum, mean, and standard deviation of the absolute and percentage errors of the neural network for the output variables.

		Minimum	Maximum	Mean	Deviation
Absolute error		3.63 × 10^−3^	0.49	0.14	0.12
Percentage error	Q1 (%)	0.14	19.08	5.54	4.71
	Q6 (%)	0.02	2.64	0.77	0.65
	Q12 (%)	0.01	1.70	0.49	0.42
	Q24 (%)	0.01	1.50	0.44	0.37
	MDT (h)	0.01	0.75	0.22	0.19

**Figure 6:**
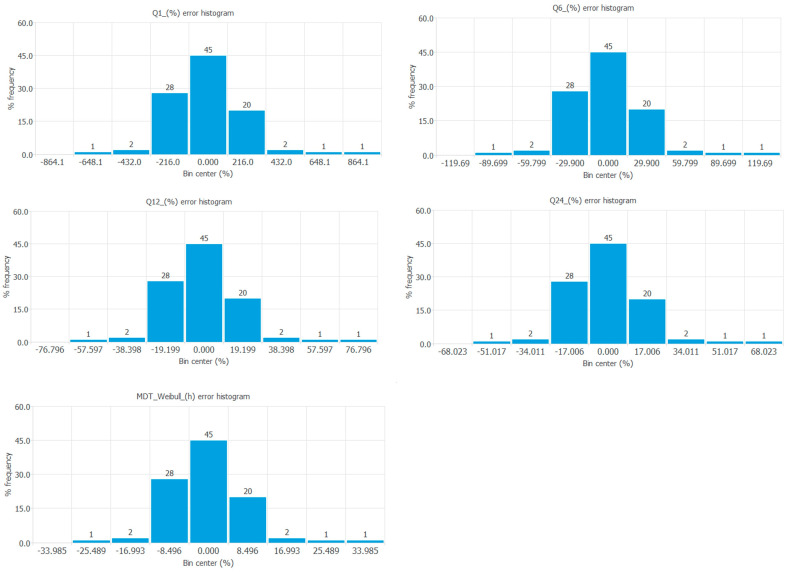
Frequency distribution of errors of the responses Q1 (%), Q6 (%), Q12 (%), Q24 (%), and mean dissolution time (h) from the Weibull equation.

The error histograms show the frequency distribution of the errors of the neural network for the output variables tested. Figure 6 shows the frequency distribution of errors of all the output variables tested.

### 3.5. Mathematical Expression of the Neural Network

The mathematical equations used for the neural network are presented in Table S2. An analysis of the standardized weights revealed the relative influence of formulation and process parameters on the model outputs (Q_1_%, Q_6_%, Q_12_%, Q_24_%, and MDT). Among the input variables, the coating agent and HPMC grade exhibited the highest absolute weight magnitudes across the hidden neurons, indicating their predominant contribution to the predicted drug release kinetics. Chitosan concentration and coating percentage also showed moderate effects, whereas mixing time had comparatively lower sensitivity, suggesting it plays a secondary role under the studied formulation space.

Overall, the ANN sensitivity pattern is consistent with the expected mechanistic influence of polymer viscosity and coating composition on the diffusion-controlled release of the drug. These findings confirm that the ANN successfully captured the nonlinear interactions between excipient composition and release behavior, as reflected by low training and selection errors (Table S2) and acceptable validation metrics.

### 3.6. Predictive Capacity of the Neural Network

The predictive capability of the neural network was assessed by comparing its outputs with the experimental target values through linear regression analysis.

**Figure 7:**
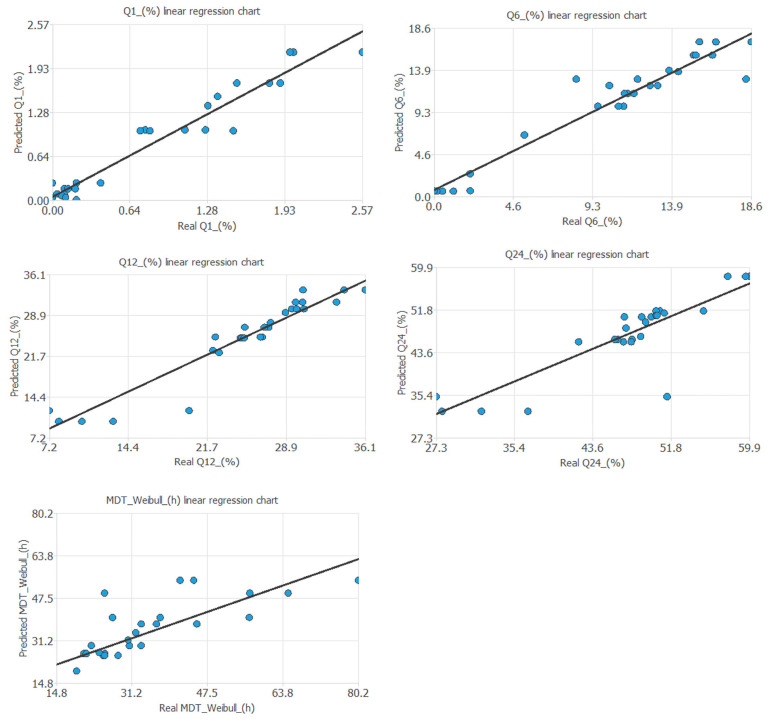
The linear relationship established between the observable and the real predicted output variables of the neural network.

**Figure 8:**
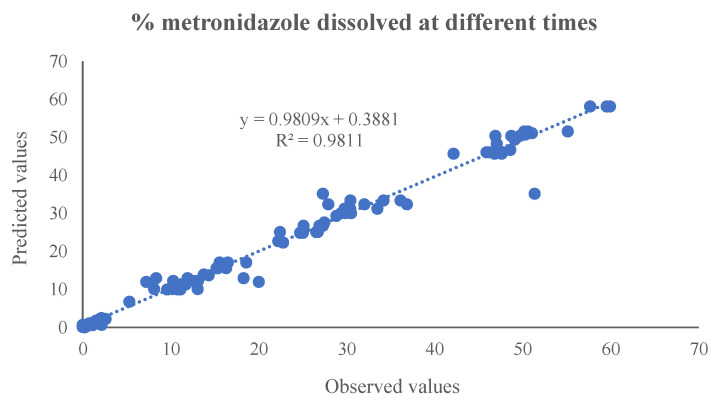
The linear relationship between the percentages of metronidazole dissolved at different times and those predicted by the neural network.

Table 2 and Figure 7 show the mathematical equations and the linear relationship established between the experimental and predicted values of the different output variables included in the artificial neural network. Figure 8 shows the linear relationship between the percentages of metronidazole dissolved at different times and the values predicted by the neural network.

Bias and precision indices (AFE, AAFE, MAE, and RMSE) for the predictions of the amount of metronidazole released at different times indicate a good predictive performance for the ANN (Table S3). A comparative study confirmed the superior predictive performance of the ANN model. The ANN was better than the Weibull model, particularly for the earlier dissolution times. Furthermore, the comparison against the Multiple Linear Regression (MLR) model confirmed the ANN’s superior predictive capacity for the later dissolution times, thereby justifying the necessity of a nonlinear approach (Table S3).

## 4. Discussion

In pharmaceutical technology, the adoption of artificial neural networks has notably increased in recent years, reflecting the growing adoption of data-driven approaches in drug development [10,21]. For instance, they have been employed in the optimization of minitablet formulations, the optimization of continuous granulation processes, the prediction of particle size in polymeric nanoparticles, and the generation of design spaces to achieve bioequivalent products under quality by design (QbD) principles, among many other applications [22,23,24,25]. The main contribution of this manuscript is the use of ANN-based artificial intelligence methods to combine information related to the formulation and manufacturing process of complex sustained-release systems with their release response. Furthermore, neural networks have allowed us to simultaneously evaluate the impact of all these factors. The model allows for integrating CPPs and CMAs to predict and optimize the formulation into the QbD framework.

The low training and validation errors, together with the small mean absolute and percentage errors observed, demonstrate the robustness of the network and its capacity for reliable generalization within the defined formulation design space. The analysis of standardized synaptic weights revealed the relative importance of individual formulation factors in governing drug release behavior.

As shown in this study, artificial neural networks allow modeling multivariate relationships between technological variables and biopharmaceutical behaviors of oral controlled-release formulations in a more effective way than conventional statistical methods, demonstrating the efficiency of in silico modeling in the optimization of new pharmaceutical dosage forms and drug delivery systems.

However, the ANN was designed to integrate multiple formulation and process variables simultaneously, allowing it to capture subtle variations within each coating category that may not be apparent from standard univariate analysis, demonstrating the efficiency of *in silico* modeling in the optimization of new pharmaceutical dosage forms and drug delivery systems.

In sustained-release pharmaceutical forms, the control of the release through the dissolution profile of the drug is essential for the control of the serum levels of the drug. This is a fundamental objective in the design of this type of pharmaceutical dosage form [26]. The ANN identified the type of coating agent, coating weight gain, and HPMC viscosity as key variables governing the dissolution profile. These findings are consistent with an established mechanistic understanding of polymer-based sustained-release systems. At early dissolution times (1–6 h), drug release was primarily controlled by the coating composition and thickness, which regulate water penetration and the onset of polymer swelling. In addition to these variables at later stages (12–24 h), the viscosity of the HPMC also influences the response of the system. High-viscosity grades generate denser gel layers that impede water penetration and diffusion, prolonging release and increasing the mean dissolution time (MDT).

The ANN exhibited low training and validation errors, narrow error distributions centered near zero, and strong correlations between observed and predicted values. These metrics confirm that the model achieved robust generalization within the experimental design space. The use of internal validation provided a reliable strategy for assessing predictive performance, especially under limited data availability.

A key reason why neural networks exhibit superior predictive performance compared with linear or semi-empirical models, such as multiple regression or the Weibull function, lies in their ability to capture nonlinear relationships, complex variable interactions, and emergent system behaviors. Unlike linear models, which assume additive and proportional effects among predictors, ANNs can model synergistic or antagonistic effects between formulation variables through their multilayered structure and nonlinear activation functions. This architecture enables them to approximate virtually any functional relationship, allowing for the identification of subtle dependencies and threshold effects that govern complex pharmaceutical systems. As a result, ANNs can describe multidimensional patterns within the formulation, process, and response space that are very limited by conventional regression approaches.

Within a QbD context, the ANN model contributes to the quantitative understanding of CMAs, CPPs, and CQAs, enabling the definition of design spaces that ensure consistent product performance. The ability to simulate how changes in individual input variables affect dissolution outcomes provides a virtual experimentation environment for risk-based optimization. This capability allows researchers to explore formulation scenarios computationally before performing confirmatory laboratory experiments, thereby reducing the development time and experimental burden.

Beyond prediction, the ANN framework serves as an interpretive tool that bridges empirical observation and mechanistic understanding. It captures the interplay between formulation composition, processing conditions, and drug release kinetics, providing a holistic picture of system behavior. Such data-driven modeling aligns with the regulatory shift toward model-informed drug development (MIDD) and supports the broader goal of integrating artificial intelligence into pharmaceutical research pipelines.

Moreover, the ANN’s adaptability enables its use as a foundation for hybrid models that combine data-driven prediction with mechanistic dissolution equations. These hybrid approaches could enhance interpretability while retaining the flexibility of machine learning, ultimately improving extrapolation to untested formulation conditions or alternative drug molecules.

Although the developed model demonstrated excellent predictive performance within the design space studied, its generalizability remains limited by the size and diversity of the experimental dataset. Future studies should incorporate external validation with independent formulations and explore transfer learning techniques to extend applicability across different drugs and polymeric systems.

## 5. Conclusions

This research demonstrates the potential of artificial neural networks as an effective complementary tool for the design and optimization of sustained-release formulations. By integrating multiple formulation and process variables, the ANN provided accurate predictions of metronidazole dissolution profiles and mechanistic insights into the roles of coating composition, polymer viscosity, and process parameters. Within the QbD paradigm, such models facilitate knowledge-driven formulation design, reduce experimental workload, and support the establishment of robust design spaces. Overall, the results highlight the growing relevance of machine learning in formulation science and its capacity to accelerate innovation in controlled-release drug delivery systems.

## Figures and Tables

**Table 2:** Mathematical equations used in the neural network for the final output from the hidden layer outputs (Yij) and the values obtained for the origin interception, slope, and correlation of the linear regression between predicted and real response values.

Equation		Intercept	Slope	Correlation
Q1 (%)	Q1 = (−0.751977 + (Y_1.1_ × 1.21766) + (Y_1.2_ × 0.393537) + (Y_1.3_ × 0.336362) + (Y_1.4_ × −0.319605))	0.046	0.942	0.971
Q6 (%)	Q6 = (0.221746 + (Y_1.1_ × −1.40995) + (Y_1.2_ × −0.416852) + (Y_1.3_ × 0.451469) + (Y_1.4_ × 0.0549343))	0.709	0.928	0.964
Q12 (%)	Q12 = (0.518512 + (Y_1.1_ × −0.282931) + (Y_1.2_ × −0.92307) + (Y_1.3_ × −0.808647) + (Y_1.4_ × −0.388919))	2.250	0.907	0.953
Q24 (%)	Q24 = (0.562707 + (Y_1.1_ × 0.254254) + (Y_1.2_ × −1.06216) + (Y_1.3_ × −1.30047) + (Y_1.4_ × −0.562973))	10.90	0.766	0.876
MDT (h)	MDT = (−0.718481 + (Y_1.1_ × −0.606845) + (Y_1.2_ × 0.956449) + (Y_1.3_ × 1.55179) + (Y_1.4_ × 0.750981))	12.70	0.621	0.789

## Data Availability

The raw data supporting the conclusions of this article will be made available by the authors on request.

## Supplementary Materials

The following supporting information can be downloaded at https://www.mdpi.com/article/10.3390/pharmaceutics17111451/s1, Table S1-A. Summary of Experimental Design Conditions (Eudragit^®^ RL 30D) [13]; Table S1-B. Summary of Experimental Design Conditions (Eudragit^®^ FS 30D) [13]; Table S2. Explicit functions obtained for the artificial neural network including the standardization of input variables, equations of hidden layer neurons (Yi,j) and the final equation of the denormalized output variables. tanh is the tangent hyperbolic activation function; Table S3. Estimated prediction errors for the amounts of metronidazole dissolved in vitro at different times (Q1, Q6, Q12 & Q24) comparing the Neural Network, Weibull and Multiple Linear Regression models.

## References

[1] Agatonovic-Kustrin S., Beresford R. (2000). Basic Concepts of Artificial Neural Network (ANN) Modeling and Its Application in Pharmaceutical Research. J. Pharm. Biomed. Anal..

[2] Kaviani S., Sohn I. (2021). Application of Complex Systems Topologies in Artificial Neural Networks Optimization: An Overview. Expert Syst. Appl..

[3] Acevedo E.S., Serna A.A., Serna E.M. (2017). Principios y Características de Las Redes Neuronales Artificiales. Desarrollo e Innovación en Ingeniería.

[4] Chen H., Engkvist O., Wang Y., Olivecrona M., Blaschke T. (2018). The Rise of Deep Learning in Drug Discovery. Drug Discov. Today.

[5] Sun Y., Peng Y., Chen Y., Shukla A.J. (2003). Application of Artificial Neural Networks in the Design of Controlled Release Drug Delivery Systems. Adv. Drug Deliv. Rev..

[6] Liang L., Guo W., Zhang Y., Zhang W., Li L., Xing X. (2020). Radial Basis Function Neural Network for Prediction of Medium-Frequency Sound Absorption Coefficient of Composite Structure Open-Cell Aluminum Foam. Appl. Acoust..

[7] Kumar P.S., Behera H., K A.K., Nayak J., Naik B. (2020). Advancement from Neural Networks to Deep Learning in Software Effort Estimation: Perspective of Two Decades. Comput. Sci. Rev..

[8] De Ramón-Fernández A., Salar-García M.J., Fernández D.R., Greenman J., Ieropoulos I.A. (2020). Evaluation of Artificial Neural Network Algorithms for Predicting the Effect of the Urine Flow Rate on the Power Performance of Microbial Fuel Cells. Energy.

[9] Paul D., Sanap G., Shenoy S., Kalyane D., Kalia K., Tekade R.K. (2021). Artificial Intelligence in Drug Discovery and Development. Drug Discov. Today.

[10] Aghajanpour S., Amiriara H., Esfandyari-Manesh M., Ebrahimnejad P., Jeelani H., Henschel A., Singh H., Dinarvand R., Hassan S. (2025). Utilizing Machine Learning for Predicting Drug Release from Polymeric Drug Delivery Systems. Comput. Biol. Med..

[11] Takayama K., Takahara J., Fujikawa M., Ichikawa H., Nagai T. (1999). Formula Optimization Based on Artificial Neural Networks in Transdermal Drug Delivery. J. Control. Release.

[12] Lefnaoui S., Rebouh S., Bouhedda M., Yahoum M.M. (2020). Artificial Neural Network for Modeling Formulation and Drug Permeation of Topical Patches Containing Diclofenac Sodium. Drug Deliv. Transl. Res..

[13] Arévalo-Pérez R., Maderuelo C., Lanao J.M. (2024). Development of Intestinal Colonic Drug Delivery Systems for Diverticular Disease: A QbD Approach. Eur. J. Pharm. Sci..

[14] Petrović J., Ibrić S., Betz G., Đurić Z. (2012). Optimization of Matrix Tablets Controlled Drug Release Using Elman Dynamic Neural Networks and Decision Trees. Int. J. Pharm..

[15] Benkő E., Ilič I.G., Kristó K., Regdon G., Csóka I., Pintye-Hódi K., Srčič S., Sovány T. (2022). Predicting Drug Release Rate of Implantable Matrices and Better Understanding of the Underlying Mechanisms through Experimental Design and Artificial Neural Network-Based Modelling. Pharmaceutics.

[16] Caccavo D. (2019). An Overview on the Mathematical Modeling of Hydrogels’ Behavior for Drug Delivery Systems. Int. J. Pharm..

[17] Baskin I.I., Winkler D., Tetko I.V. (2016). A renaissance of neural networks in drug discovery. Expert Opin. Drug Discov..

[18] Sharma R., Singh D., Gaur P., Joshi D. (2021). Intelligent Automated Drug Administration and Therapy: Future of Healthcare. Drug Deliv. Transl. Res..

[19] Mahmood I., Tegenge M.A. (2019). A Comparative Study Between Allometric Scaling and Physiologically Based Pharmacokinetic Modeling for the Prediction of Drug Clearance from Neonates to Adolescents. J. Clin. Pharmacol..

[20] Artelnics (2022). Neural Designer, version 4.

[21] Leite M.L., Costa L.S.d.L., Cunha V.A., Kreniski V., Filho M.d.O.B., da Cunha N.B., Costa F.F. (2021). Artificial Intelligence and the Future of Life Sciences. Drug Discov. Today.

[22] Barmpalexis P., Karagianni A., Karasavvaides G., Kachrimanis K. (2018). Comparison of Multi-Linear Regression, Particle Swarm Optimization Artificial Neural Networks and Genetic Programming in the Development of Mini-Tablets. Int. J. Pharm..

[23] Shirazian S., Zeglinski J., Darwish S., Kuhs M., Albadarin A.B., Croker D.M., Walker G.M. (2018). Continuous Twin Screw Wet Granulation: The Combined Effect of Process Parameters on Residence Time, Particle Size, and Granule Morphology. J. Drug Deliv. Sci. Technol..

[24] Youshia J., Ali M.E., Lamprecht A. (2017). Artificial Neural Network Based Particle Size Prediction of Polymeric Nanoparticles. Eur. J. Pharm. Biopharm..

[25] Simões M.F., Silva G., Pinto A.C., Fonseca M., Silva N.E., Pinto R.M.A., Simões S. (2020). Artificial Neural Networks Applied to Quality-by-Design: From Formulation Development to Clinical Outcome. Eur. J. Pharm. Biopharm..

[26] Bannigan P., Aldeghi M., Bao Z., Häse F., Aspuru-Guzik A., Allen C. (2021). Machine Learning Directed Drug Formulation Development. Adv. Drug Deliv. Rev..

