# Supplementary material for: Building Artificial Neural Networks for the Optimization of Sustained-Release Kinetics of Metronidazole from Colonic Hydrophilic Matrices
Source: Pharmaceutics. 2025 Nov 10;17(11):1451. doi: 10.3390/pharmaceutics17111451 (PMC12655049; doi:10.3390/pharmaceutics17111451)
Supplement: Supplementary file 1 [file pharmaceutics-17-01451-s001.zip › pharmaceutics-3887456-supplementary.pdf]

Supplementary material

Building Artificial Neural Networks for the Optimization of Sustained-Release Kinetics of Metronidazole from Colonic Hydrophilic Matrices

Cristina Maderuelo [1,2], Roberto Arévalo-Pérez [1] and José M. Lanao [1, 2,*]

[1] Area of Pharmacy and Pharmaceutical Technology, Department of Pharmaceutical Sciences, Faculty of Pharmacy, University of Salamanca, 37007 Salamanca, Spain.
[2] Institute of Biomedical Research of Salamanca (IBSAL), 37007 Salamanca, Spain
* Correspondence: jmlanao@usal.es

Table S1-A. Summary of Experimental Design Conditions (Eudragit® RL 30D) [13].

Batch	HPMC Grade	% HPMC	% CH	Blending time (min)	% Δ W
F1, F18, F22	K15	8	24	20	10
F8, F9, F15	K35	24	8	10	10
F6, F7, F12	K15	8	24	10	20
F2, F20, F25	K35	24	8	20	20
F21	K15	16	16	15	15
F14	K35	16	16	15	15

Table S1-B. Summary of Experimental Design Conditions (Eudragit® FS 30D) [13].

Batch	HPMC Grade	% HPMC	% CH	Blending time (min)	% Δ W
F4, F5, F11	K15	24	8	20	10
F13, F16, F17	K35	8	24	10	10
F3, F23, F27	K15	24	8	10	20
F19, F26, F28	K35	8	24	20	20
F24	K15	16	16	15	15
F10	K35	16	16	15	15

[13] Arévalo-Pérez, R.; Maderuelo, C.; Lanao, J.M. Development of Intestinal Colonic Drug Delivery Systems for Diverticular Disease: A QbD Approach. Eur. J. Pharm. Sci. 2024, 203, 106918, doi:10.1016/j.ejps.2024.106918.

Table S2. Explicit functions obtained for the artificial neural network including the standardization of input variables, equations of hidden layer neurons (Yi,j) and the final equation of the denormalized output variables. tanh is the tangent hyperbolic activation function.

Standardization of input variables	
**HPMC grade**	HPMC grade mPa.s = (HPMC grade – 25000)/10183.5
**HPMC (%)**	HPMC % = (HPMC % - 15.9)/7.50006
**Chitosan (%)**	Chitosan % = (Chitosan % - 15.9)/7.50006
**Mixing Time (min)**	Mixing Time min = (Mixing Time min - 15)/4.71405
**Coating Agent**	Coating Agent = (Coating Agent - 1.5)/0.509175
**% Coating**	% coating = (% coating - 15)/4.71405
**Equations of hidden layer neurons**	
$\mathbf{Y_{1.1}}$ = tanh (-0.122154 + (HPMC grade*0.0169062) + (HPMC % *-0.398129) + (Chitosan %*-0.50438) + (Mixing Time*-0.0579495) + (Coating Agent*-0.944573) + (% coating*0.331476))	
$\mathbf{Y_{1.2}}$ = tanh (1.3557 + (HPMC grade*-0.286625) + (HPMC %*-0.031695) + (Chitosan %*0.0315847) + (Mixing Time*0.229054) + (Coating Agent*1.26565) + (% coating*0.851109))	
$\mathbf{Y_{1.3}}$ = tanh (-0.87853 + (HPMC grade*1.02149) + (HPMC %*0.591327) + (Chitosan_ %*-0.531824) + (Mixing Time*-0.338266) + (Coating Agent*-2.36414) + (%_coating*-0.177376))	
$\mathbf{Y_{1.4}}$ = tanh (-1.02566 + (HPMC grade*0.17873) + (HPMC %*-0.327629) + (Chitosan %*0.305227) + (Mixing Time*0.137167) + (Coating Agent*1.49789) + (%_coating*0.752126))	
**Equation of the denormalized output variables**	
**(Q1 %, Q6 %, Q12 %, Q24 %, MDT h)** = (0.5*(Q1 % + 1.0)*(2.57-0) + 0,0.5*(Q6 % + 1.0)*(18.57-0) + 0,0.5*(Q12 % + 1.0)*(36.13 - 7.21) + 7.21,0.5*(Q24 % + 1.0)*(59.93 - 27.28) + 27.28,0.5*(MDT h + 1.0)*(80.17-14.82) + 14.82)	

Table S3. Estimated prediction errors for the amounts of metronidazole dissolved *in vitro* at different times (Q1, Q6, Q12 & Q24) comparing the Neural Network, Weibull and Multiple Linear Regression models.

Error	Prediction model	Q1	Q6	Q12	Q24
**AFE**	Neural Network	0.86	1.00	1.02	1.01
	Weibull	0.25	1.00	1.01	0.99
	MLR	1.24	1.02	1.02	1.00
**AAFE**	Neural Network	1.48	1.24	1.11	1.61
	Weibull	4.52	1.00	1.34	1.17
	MLR	1.48	1.27	1.14	1.11
**MAE (%)**	Neural Network	0.14	1.01	1.61	2.49
	Weibull	0.71	3.50	5.74	6.73
	MLR	0.17	1.16	2.38	4.22
**RMSE (%)**	Neural Network	0.19	1.58	2.42	4.18
	Weibull	1.04	4.63	7.34	8.62
	MLR	0.21	1.82	2.96	5.38
